# Examining the Relationship between Nighttime Glucose Values in Youth with Type 1 Diabetes and Parent Fear of Nighttime Hypoglycemia

**DOI:** 10.1155/2023/9953662

**Published:** 2023-04-14

**Authors:** Alexandra D. Monzon, Ryan McDonough, Christopher C. Cushing, Mark Clements, Susana R. Patton

**Affiliations:** ^1^Nemours Children's Health, Nemours Center for Healthcare Delivery Florida, Orlando, FL, USA; ^2^University of Kansas, Clinical Child Psychology Program, Lawrence, KS, USA; ^3^Children's Mercy Hospitals and Clinics, Pediatric Endocrinology, Kansas City, MO, USA; ^4^Nemours Children's Health, Nemours Center for Healthcare Delivery Florida, Jacksonville, FL, USA

## Abstract

**Objective:**

Youth with type 1 diabetes mellitus (T1D) are at risk for experiencing nighttime hypoglycemia, and many parents report significant anxiety at night regarding glucose management. Limited data exist examining continuous nighttime glucose levels as a predictor of parent fear of nighttime hypoglycemia. The present study aimed to examine the relationship between parent fear of nighttime hypoglycemia and nighttime blood glucose levels as measured by continuous glucose monitors (CGMs).

**Methods:**

A sample of 136 parents/caregivers of youth with T1D completed a one-time survey and youth provided 14 days of CGM data. We conducted regression models with mean nighttime glucose value, glycemic variability, and the percent of nighttime glucose values in the hypoglycemic, target, and hyperglycemic range as the independent variable and parents' fear of nighttime hypoglycemia as the dependent variable.

**Results:**

Overnight hypoglycemia measured via CGM did not predict parents' fear of nighttime hypoglycemia; however, average youth nighttime glucose levels and nighttime glycemic variability were significant predictors of parents' fear of nighttime hypoglycemia.

**Conclusions:**

The results of the present study indicate that parents of youth with T1D may report higher fear of hypoglycemia if they observe increased fluctuations in their child's nighttime glucose levels, regardless of how often their child's glucose levels are in the hypoglycemic range. The results suggest that clinicians may consider screening for parent fear of nighttime hypoglycemia in families of youth who present with large variability in their glucose values overnight.

## 1. Introduction

Instances of hypoglycemia (i.e., glucose <70 mg/dL or 3.9 mmol/L) are a common and potentially dangerous complication of type 1 diabetes (T1D) insulin management. It is common for parents of youth with T1D to report elevated stress and anxiety regarding nighttime caregiving [1]. In fact, parents of youth with T1D report that overnight glucose management is the most challenging aspect of T1D care, and that nighttime is when they worry the most about their child's blood glucose levels [2]. This concern often leads parents to regularly monitor their child's blood glucose concentrations throughout the night [1]. Parents may monitor their child's glucose levels by obtaining a fingertip blood sample for a blood glucose meter or by using a continuous glucose monitor (CGM), where a sensor is placed under the skin and glucose levels are monitored via smartphone or small device [3]. The term “fear of nighttime hypoglycemia” is used to describe the worry and avoidance behaviors persons with T1D, or their caregivers, may experience related to nighttime low glucose levels. While it is necessary for caregivers to provide appropriate vigilance with glucose management to obtain optimal glycemic control, previous studies indicate that when worries of low blood glucose intensify to significant anxiety, caregivers endorse substantial distress and reduced quality of life, and their child may experience higher glycemic levels, as measured by glycated hemoglobin A1C (A1C; [4–9]).

Previous studies indicate the most common predictor of parent fear of hypoglycemia is self-reported incidents of past hypoglycemic episodes [10, 11]. Yet, one striking omission from the current literature examining predictors of parental fear of hypoglycemia is a study examining the influence of youth nighttime glycemia based on objective data captured continuously via CGMs. Previous studies have mainly relied on A1C as a measure for child glycemic control when examining the relationship between glucose patterns and parent fear of nighttime hypoglycemia [7, 12]. Unfortunately, A1C does not provide any information regarding a person's glucose variability and does not allow for a nuanced examination of how parent nighttime fear may relate to youth's actual nighttime glycemic pattern (e.g., time below target and time above target).

The aim of the present study is to fill a gap in the literature and examine the relationship between a child's nighttime glucose values, using continuous glucose data from CGMs, and their parent's fear of nighttime hypoglycemia in families of youth with T1D. First, we examined if nighttime hypoglycemia predicted parents' fear of nighttime hypoglycemia and hypothesized that parents of youth with a higher percentage of nighttime glucose levels in the hypoglycemia range (glucose <70 mg/dL) would report higher fear of nighttime hypoglycemia. Next, we examined if other glycemic metrics (i.e., average glucose level and glycemic variability) predicted parents' fear of nighttime hypoglycemia and hypothesized that parents of youth with higher average nighttime glucose levels and/or greater variability in nighttime glucose levels would report higher fear of nighttime hypoglycemia. Lastly, as an exploratory analysis, we examined if parents' fear of nighttime hypoglycemia or their use of hypoglycemia avoidance behaviors predicted youth nighttime hyperglycemia. The results of the present study may provide the necessary information to guide clinical care and research regarding parent fear of nighttime hypoglycemia among youth with T1D.

## 2. Methods

### 2.1. Participants

We recruited parents/caregivers of youth with T1D from diabetes clinics at a large stand-alone tertiary pediatric hospital system in the Midwestern United States. Eligibility criteria included parents of youth who (1) were between the ages of 8–17 years, (2) had a confirmed diagnosis of T1D for at least 6 months, (3) were on intensive insulin therapy (i.e., multiple daily injections and insulin pump), (4) used a personal CGM, and (5) parents who could read and comprehend English. Exclusion criteria included parents of youth who (1) had type 2 or monogenic diabetes, (2) were currently using corticosteroids, and (3) were currently using a low glucose suspend insulin pump (LGSP) or automated insulin delivery (AID) system. While it is not known if the ability to automatically reduce or suspend insulin in response to a low glucose event would have any impact on parents' fear of hypoglycemia, at the time of the study, there was limited local uptake (18% of the recruitment sample) of LGSP and AID technology, leading to the concern that there might not be enough youth on it to allow for follow-up sensitivity analyses.

### 2.2. Procedures

We submitted all study procedures to the institutional review board for approval before recruitment. We identified eligible participants using electronic health records and contacted potentially eligible participants by phone to explain the study procedures and seek informed consent for all participating parents and assent for their children. Once enrolled, we sent participants a one-time REDCap survey [13] with questions relating to fear of hypoglycemia. In addition, we asked parents to ensure their child was wearing their CGM continuously over a 14-day period coinciding with when parents completed the survey. Parents completed the REDcap survey one night in the middle of this 14-day monitoring period (i.e., day 7) before their child went to bed. Parents also reported their child's bedtime and wake time to define each child's nighttime glucose levels. We collected CGM data through the pediatric diabetes center after parents had shared the data with the clinic or had them downloaded during a routine quarterly endocrinology visit. We provided a $15 electronic gift card to all participating families.

### 2.3. Measures

#### 2.3.1. Demographics and Medical History

All parents completed information on family demographics (e.g., age and socioeconomic status) and their child's medical history (e.g., date of diagnosis, use of T1D devices, and history of severe hypoglycemia events). We conducted an electronic medical record review to obtain the child or adolescent's most recent A1C.

#### 2.3.2. Continuous Glucose Monitor (CGM)

Children and adolescents wore their own CGM device as a continuous measure of their glucose levels for the 14-day study period. We calculated the percent of glucose values in the hypoglycemic range (i.e., glucose <70 mg/dL), the percent of glucose values within the target range (i.e., glucose 70–180 mg/dL), the percent of glucose values in the hyperglycemic range (i.e., glucose >180 mg/dL), the coefficient of variation (i.e., (standard deviation/mean) *∗* 100), and the mean and standard deviation for all nighttime glucose levels. We selected these metrics because they are components of the ambulatory glucose profile that is often used in clinical decision-making, and consensus guidelines suggest that these metrics be collected in all type 1 diabetes-related clinical trials [14].

#### 2.3.3. Fear of Hypoglycemia

Parents completed the Hypoglycemia Fear Survey for Parents (HFS-P; [15]). The HFS-P is a 25-item measure that contains a “Behavior” scale, which assesses behaviors parents may engage in to keep their child from becoming hypoglycemic, and a “worry” scale, which assesses anxiety regarding the negative consequences of hypoglycemia. The Behavior scale can be further broken down into a 4-item “maintain high blood glucose (BG)” subscale which represents actions that maintain higher glucose levels [16]. For each item, parents rated how frequently each statement was true for them on a 5-point Likert scale (e.g., from “never” to “very often”) with higher scores indicating more behaviors to increase glucose levels. We only used the maintain high BG subscale in the present study for the exploratory analysis due to evidence suggesting an association between parent fear of hypoglycemia avoidance behaviors and higher glycemic levels [7, 17]. Cronbach's alpha for the maintain high BG subscale in the present sample was 0.84.

#### 2.3.4. Fear of Nighttime Hypoglycemia

To assess parent fear of nighttime hypoglycemia more comprehensively, our research group created 15 new items that assess specific worries related to nighttime hypoglycemia [18]. This “Nighttime Worry” subscale was designed to be included with the HFS-P as an additional subscale. Like the original HFS-P items, parents rated how frequently each Nighttime Worry subscale item was true for them on a 5-point Likert scale (e.g., from “never” to “very often”). Higher scores on the subscale indicate higher fear of nighttime hypoglycemia. The Nighttime Worry subscale evidenced high internal consistency with Cronbach's alpha of 0.96.

### 2.4. Data Analytic Plan

We used CGM data to calculate the percentage of glucose values in the hypoglycemic range (i.e., glucose <70 mg/dL), within the target range (i.e., glucose 70–180 mg/dL), and in the hyperglycemic range (i.e., glucose >180 mg/dL) each night during the 14-day period. We calculated each child's nighttime glucose levels based on daily reported bedtime and wake time. We then aggregated item scores on the Nighttime Worry subscale to create a total score and then conducted a regression model with the child's percent of glucose values in the hypoglycemic range as the independent variable and the parents' Nighttime Worry score as the dependent variable. For the secondary analysis, we used the coefficient of variation, mean, and standard deviation of daily glucose levels as predictors in the regression models to predict scores on the Nighttime Worry subscale. Lastly, as an exploratory analysis, we aggregated item scores on the HFS-P maintain high BG subscale to obtain a total score and conducted a regression model with the parents' HFS-P maintain high BG score as the independent variable and the child's percent of glucose values in the hyperglycemic range as the dependent variable.

## 3. Results

We recruited a sample of 165 parents/caregivers of youth with T1D. Of the 165 caregivers recruited, 136 completed the REDCap survey. See Table 1 for sample characteristics of participants. We were able to acquire CGM data from 116 children and adolescents (85.3%) of the 136 caregivers who completed the REDCap survey. The average nighttime glucose value was 181 mg/dL (SD = 49.3), and the average coefficient of variation was 33.8% (SD = 8.7). Overnight, approximately 53.6% of glucose values were in the target range, 2.3% were in the hypoglycemia range, and 44.1% were in the hyperglycemia range across the sample. See Table 2 for average daytime and nighttime glycemic levels. No demographic variables significantly correlated with the Nighttime Worry score (i.e., child age and family income). Nor did we observe group differences between insulin regimen, child sex, or parent sex on Nighttime Worry scores. The Nighttime Worry score did significantly positively correlate with parent-reported frequency of treating low glucose events at night during the study period (*r* = 0.34, *p* < 0.05), such that parents reporting more frequent treatment of nighttime low glucose also reported higher fear of hypoglycemia. Because of this association, we controlled for parent-reported frequency of treating low glucose at night in subsequent analyses. See Table 3 for correlations between Nighttime Worry scores and glycemic metrics.

We examined if the percent of youth's overnight glucose values in the hypoglycemic range predicted parents' responses on the Nighttime Worry subscale. While the regression model for the Nighttime Worry subscale was significant *F*(1, 115) = 4.20 and *p* < 0.05, the effect was more related to parents providing treatment for low glucose at night (*β* = 0.23 and *p* < 0.01) and not related to the percent of youth's glucose values in the hypoglycemic range (*β* = 0.09 and *p*=0.35). We then repeated the same regression models using youth's mean nighttime glucose values as the predictor, which significantly predicted parent responses on the Nighttime Worry scale *F*(1, 115) = 5.68 and *p* < 0.01 while controlling for the frequency of parent treatment of nighttime low glucose (*β* = 0.17 and *p* < 0.05). We then examined the standard deviation of nighttime glucose values, or glycemic variability, as the predictor, which revealed a significant regression model for parent responses on the Nighttime Worry subscale *F*(1, 115) = 7.50 and *p* < 0.01 while controlling for the frequency of parent treatment of nighttime low glucose (*β* = 0.22 and *p* < 0.05). We then examined the coefficient of variation as the predictor. The regression model for the Nighttime Worry subscale *F*(1, 115) = 4.20 and*p* < 0.05 was significant, though the effect was more related to parents providing treatment for low glucose at night (*β* = 0.23 and *p* < 0.05) and not related to percent of youth's glucose values in the hypoglycemic range (*β* = 0.14 and *p*=0.13).

As an exploratory analysis, we conducted a regression model examining if parents' Nighttime Worry score predicted youth's percent of glucose values in the hyperglycemic range overnight. The only demographic variable that significantly correlated with nighttime glucose values in the hyperglycemic range was age at diagnosis (*r* = −0.29 and *p* < 0.01), suggesting youth who were diagnosed with T1D at a younger age were more likely to have more nighttime glucose values in the hyperglycemic range. Therefore, we controlled for this variable in the exploratory analyses. The model predicting the percent of glucose values in the hyperglycemic range was statistically significant *F*(1, 115) = 6.13 and *p* < 0.05; however, the effect was more related to age at diagnosis (*β* = −0.27 and *p* < 0.01) and not related to parents' Nighttime Worry scores (*β* = 0.13 and *p*=0.14). As an additional exploratory analysis, we examined a regression model assessing if parents' HFS-P maintain high BG scores predicted youth's percent of glucose values in the hyperglycemic range. The model predicting percent of glucose values in the hyperglycemic range was statistically significant *F*(1, 115) = 9.86 and *p* < 0.01 such that parents' maintain high BG scale score significantly predicted the proportion of nighttime glucose values that were in the hyperglycemic range (*β* = 0.27 and *p* < 0.01), even after controlling for youth's age of T1D diagnosis. See Table 3 for correlations between HFS-P maintain high BG subscale scores and glycemic metrics.

## 4. Discussion

This current study is the first to examine the association between parental fear of nighttime hypoglycemia and youth's continuous nighttime glucose patterns. Contrary to the primary hypothesis, youth's percentage of nighttime hypoglycemic glucose levels (i.e., <70 mg/dl) did not significantly predict their parent's score on the Nighttime Worry scale. In support of the secondary hypothesis, youth's mean nighttime glucose values did significantly predict their parent's Nighttime Worry scores, and the standard deviation of youth's nighttime glucose values also significantly predicted their parents' Nighttime Worry scores. However, in the exploratory analyses, while youth's percentage of nighttime hyperglycemic glucose levels (i.e., >180 mg/dl) did not predict their parents' Nighttime Worry scores, we did observe a significant association between youth's percentage of nighttime hyperglycemic glucose levels and parents' Maintain High BG score.

We perceive two possible reasons for why the study results did not support the primary hypothesis. First, it is possible there was no support because our sample had a low rate of nighttime hypoglycemia (*M* = 2.3%). This low rate would reduce the overall variability of glucose levels in the models and possibly lead to a type 2 error. Even so, our rate of nighttime hypoglycemia appears consistent with at least one study which reported nighttime hypoglycemic events occurring in less than 10% of nights in older youth and adolescents with T1D over a year-long period [19]. Thus, we do not think our rate of nighttime hypoglycemia was unusually low for youth. Second, it is possible that there was no support because 60% of the youth in our sample used an insulin pump. Recent data from the Type 1 diabetes exchange suggest an insulin pump rate of 63% among youth in the United States (US), indicating the pump rate in our sample is representative of youth in the US [20]. However, some evidence suggests an association between pump therapy and lower rates of severe hypoglycemia [21], which in turn could reduce parents' fear of hypoglycemia overall and the power of our models to detect an association between youth percentage of nighttime hypoglycemic glucose levels (i.e., <70 mg/dl) and parents' fear of nighttime hypoglycemia. Of note, in our analyses, we did not find a significant difference in parents' Nighttime Worry scores based on youth insulin regimen, though one previous study has found a difference in parent-reported fear of nighttime hypoglycemia and child insulin regimen [22]. It is possible we did not replicate the previous results because our sample was comprised of older youth, and we used a comprehensive measure of parents' fear of nighttime hypoglycemia versus a single-item measure. Yet, future research should further explore this relationship.

There was support found for the secondary hypothesis of the study, which examined the associations between other youth nighttime glucose metrics and parents' nighttime fear of hypoglycemia. Specifically, we found that higher youth mean nighttime glucose levels predicted higher parent fear of hypoglycemia at night. We also found that higher youth variability in nighttime glucose levels predicted higher parent fear of nighttime hypoglycemia. Youth's mean nighttime glucose value was 181 mg/dL (SD = 49.3) and youth averaged 53.53% time in range overnight. Therefore, it is possible that the association between youth's mean nighttime glucose levels and parents' report of nighttime fear may suggest that for some parents, even glucose values in the target range (i.e., 70–180 mg/dl) feel low and exacerbate their fear. This would support previous studies that examined the association between parents' report of daytime fear of hypoglycemia and children's glucose levels. The association between youth's nighttime glycemic variability and parents' Nighttime Worry scores may suggest that parents worry more when their child experiences large fluctuations in their nighttime glucose levels perhaps because they anticipate an increased possibility their child could experience a hypoglycemic event. As a reminder, the current study excluded youth using AID or LGSP, which are technologies that have the functionality to automatically reduce or suspend basal insulin if a low glucose level is detected/predicted. There is recent data from a trial initiating LGSP in youth with T1D showing a reduction in parent general fear of hypoglycemia and improvements in youth's mean nighttime glucose levels, standard deviation of nighttime glucose levels, and percentage of in range glucose levels (i.e., 70–180 mg/dl) with LGSP [23]. However, despite these very promising results, the trial reported no reductions in instances of nighttime hypoglycemia and potentially significant increases in nighttime instances of severe hypoglycemia [23] among youth using the LGSP. Because of these mixed findings, it may be helpful for a future study to re-test the associations from the secondary hypothesis in a sample of youth using an AID or LGSP and using a measure that focuses specifically on parents' nighttime fear.

Interestingly, while exploratory analyses did not show an association between parents' Nighttime Worry and youth's percentage of nighttime hyperglycemic levels, the association between parents' Maintain High BG score and youth's percent of nighttime hyperglycemic levels could indicate a tendency among parents with greater fear to engage in hypoglycemia avoidance behaviors that may affect youth's nighttime glucose. This finding is consistent with previous studies that show an association between parent fear of hypoglycemia and higher A1C levels in youth [7, 17], though we believe our use of CGM data to measure youth's glycemia and our focus on nighttime glucose pattern provide new data that extend the literature.

The study results have implications for providers working with youth with T1D and their families. First, clinicians may consider screening for parental fear of nighttime hypoglycemia to further determine if nighttime glucose management should be a target for diabetes education. For instance, clinicians may wish to assess for parental fear of nighttime hypoglycemia in clinic and discuss parents' behaviors that maintain high glucose levels if youth are experiencing frequent or prolonged periods of nighttime hyperglycemia. Second, clinicians may consider offering parents strategies to manage their specific worries related to nighttime hypoglycemia, especially if these fears begin to negatively impact quality of life, caregiver sleep, or parent-child conflict. Parent-focused interventions that utilize cognitive behavioral strategies exist and have demonstrated significant reductions in caregiver fear of hypoglycemia and child A1C [24]. To date, most studies examining parent fear of hypoglycemia are cross-sectional indicating a need for longitudinal studies to assess important developmental trends in parent fear of hypoglycemia following a T1D diagnosis. Longitudinal studies may also inform recommendations advising on the frequency at which to screen for parent fear of hypoglycemia and when to intervene with parents.

There are some limitations to consider when interpreting the results from the current study. The participant demographics in the current study are representative of the overall patient population at the recruiting hospital system [25, 26] but may not fully generalize to underrepresented groups more globally. Disparities exist among youth with T1D regarding insulin treatment methods and overall glycemic control [27, 28] suggesting that multisite studies that span more than one geographic region are needed to better understand how fear of nighttime hypoglycemia relates to youth nighttime glucose patterns in more demographically diverse samples. Furthermore, the inclusion criteria limited the study sample to parents of youth actively using a CGM device and excluded youth using LGSP or self-monitoring blood glucose via fingerstick. Each method of glucose monitoring may differentially affect fear of nighttime hypoglycemia and remains an area for future study. Lastly, the sample of youth in the current study demonstrated lower glycemic levels than typically observed in school-aged children and adolescents with T1D. Currently, the American Diabetes Association recommends an A1C value of 7% or lower for most youth with T1D [29], and across all participants, 31% of youth met this glycemic target. National registry data suggest that approximately 17% of youth with T1D under the age of 18 years met the previously recommended A1C value of 7.5% [20], indicating that the overall study sample may not accurately reflect national glycemic patterns in youth with T1D. The CGM inclusion criteria for the current study and high insulin pump use among youth in the study may partially account for the A1C values observed in the current sample.

## 5. Conclusions

The results of the present study suggest that parents of youth with T1D may report higher fear of nighttime hypoglycemia if they observe increased fluctuations in their child's nighttime glucose levels, regardless of the percent of time their child's glucose levels are in the hypoglycemic range. These results have important implications for parents who may engage in behaviors that maintain high glucose levels and may require additional T1D education or support to reduce symptoms of anxiety. These findings are important to clinicians who may consider screening for parent fear of nighttime hypoglycemia and researchers interested in examining the relationship between parent fear of nighttime hypoglycemia and the effectiveness of new T1D treatment devices.

## Figures and Tables

**Table 1:** Sample characteristics of participants.

Parent/child (*n* = 136) characteristics	Mean ± SD	*n* (%)
Age (years)	41.76 ± 6.52	
Caregiver (mother)		121 (89)
Household income (>$80,000)		81 (60)
Caregiver relationship status (married or living with partner)		108 (79)
Caregiver race/ethnicity		
Nonhispanic White		125 (92)
Hispanic White		3 (2)
Black or African American		6 (4)
Native or Indigenous American		1 (<1)
Hawaiian or Pacific Islander		1 (<1)
Bi-racial		1 (<1)
Child age (years)	13.41 ± 2.59	
Child duration of T1D (years)	3.97 ± 3.14	
Child A1C	7.93 ± 1.72	
Child A1C > 7.0%		94 (69)
Child insulin pump use		81 (60)
Child biological sex (female)		66 (49)
Child race/ethnicity		
Nonhispanic White		111 (82)
Hispanic White		4 (3)
Black or African American		8 (6)
Native or Indigenous American		3 (2)
Hawaiian or Pacific Islander		1 (<1)
Bi-racial		9 (6)

**Table 2:** Average youth daytime and nighttime glycemic levels.

CGM metric	Daytime		Nighttime	
	*M* ± SD	%	*M* ± SD	%
Glucose level	187.99 ± 51.04		184.44 ± 49.31	
Coefficient of variation	35.68 ± 6.91		33.83 ± 8.66	
Percent time in range		51.94		53.54
Percent time in hyperglycemia		45.47		44.06
Percent time in hypoglycemia		2.49		2.29

**Table 3:** Correlations between nighttime worry and HFS-P maintain high BG subscale scores and glycemic metrics.

Glycemic metric	Nighttime worry score	HFS-P maintain high BG
Nighttime glucose		
Average glucose level	0.15	0.27^*∗∗*^
Glycemic variability	0.24^*∗*^	0.30^*∗∗*^
Coefficient of variation	0.18	0.13
Percent time in range	−0.16	−0.31^*∗∗*^
Percent time in hyperglycemia	0.16	0.31^*∗∗*^
Percent time in hypoglycemia	−0.05	−0.06
Daytime glucose		
Average glucose level	0.22^*∗*^	0.31^*∗∗*^
Glycemic variability	0.20^*∗*^	0.30^*∗∗*^
Coefficient of variation	0.09	0.08
Percent time in range	−0.24^*∗∗*^	−0.33^*∗∗*^
Percent time in hyperglycemia	0.23^*∗*^	0.34^*∗∗*^
Percent time in hypoglycemia	−0.07	−0.23^*∗*^

*Note*. ^*∗*^Significant at the 0.05 level. ^*∗∗*^Significant at the 0.01 level.

## Data Availability

The data used to support the findings of this study are available from the corresponding author upon request.
